# Incidence and Simple Prediction Model of Hyperuricemia for Urban Han Chinese Adults: A Prospective Cohort Study

**DOI:** 10.3390/ijerph14010067

**Published:** 2017-01-11

**Authors:** Jin Cao, Chunxia Wang, Guang Zhang, Xiang Ji, Yanxun Liu, Xiubin Sun, Zhongshang Yuan, Zheng Jiang, Fuzhong Xue

**Affiliations:** 1 Department of Biostatistics, School of Public Health, Shandong University, Jinan 250012, Shandong, China; caojin0806@163.com (J.C.); liu-yx@sdu.edu.cn (Y.L.); sunxiubin@sdu.edu.cn (X.S.); yuanzhongshang@sdu.edu.cn (Z.Y.); lawjiang1@hotmail.com (Z.J.); 2 Health Management Center, Affiliated Hospital of Jining Medical University, Jining 272000, Shandong, China; jyfy_tjzx@163.com; 3 Health Management Center, Shandong Provincial Qianfoshan Hospital, Jinan 250014, Shandong, China; zhangguangsd@163.com; 4 Geriatrics Qilu Hospital of Shandong University, Jinan 250012, Shandong, China; drjixiang@126.com

**Keywords:** hyperuricemia, cohort study, predictive model, Cox model, gender specific

## Abstract

Background: Hyperuricemia (HUA) contributes to gout and many other diseases. Many hyperuricemia-related risk factors have been discovered, which provided the possibility for building the hyperuricemia prediction model. In this study we aimed to explore the incidence of hyperuricemia and develop hyperuricemia prediction models based on the routine biomarkers for both males and females in urban Han Chinese adults. Methods: A cohort of 58,542 members of the urban population (34,980 males and 23,562 females) aged 20–80 years old, free of hyperuricemia at baseline examination, was followed up for a median 2.5 years. The Cox proportional hazards regression model was used to develop gender-specific prediction models. Harrell’s C-statistics was used to evaluate the discrimination ability of the models, and the 10-fold cross-validation was used to validate the models. Results: In 7139 subjects (5585 males and 1554 females), hyperuricemia occurred during a median of 2.5 years of follow-up, leading to a total incidence density of 49.63/1000 person years (64.62/1000 person years for males and 27.12/1000 person years for females). The predictors of hyperuricemia were age, body mass index (BMI) systolic blood pressure, serum uric acid for males, and BMI, systolic blood pressure, serum uric acid, triglycerides for females. The models’ C statistics were 0.783 (95% confidence interval (CI), 0.779–0.786) for males and 0.784 (95% CI, 0.778–0.789) for females. After 10-fold cross-validation, the C statistics were still steady, with 0.782 for males and 0.783 for females. Conclusions: In this study, gender-specific prediction models for hyperuricemia for urban Han Chinese adults were developed and performed well.

## 1. Background

Uric acid is the end product of purine metabolism in human beings. Hyperuricemia is a set of heterogeneity diseases caused by obstacles in purine metabolism and/or a decrease in the excretion of uric acid. Nowadays, hyperuricemia is becoming a serious public health problem. According to the US National Health and Nutrition Examination Survey 2007–2008 study, the prevalence of hyperuricemia was over 21% in both males and females [1]. With economic development and the change of people’s lifestyles in China, the prevalence of hyperuricemia is also increasing rapidly, having reached up to 13.7% (21% in males and 7.9% in females) during 2008–2010 in northern and northeastern Chinese provinces [2], and the prevalence is expected to rise substantially over the next few decades.

The onset of hyperuricemia is associated with many adverse health outcomes; various studies demonstrated that the incidence of hyperuricemia will increase the risk of many common diseases such as gout, renal damage, components of metabolic syndrome (MetS), cardiovascular disease (CVD), carotid atherosclerosis, etc. [3,4,5,6,7,8]. The most serious sequela of hyperuricemia is gout. It reported that 18.8% of the patients with hyperuricemia developed gout in a five-year follow-up study [9]. Cohort studies in the Netherlands and Taiwan showed that hyperuricemia was associated with metabolic syndrome and was an independent risk factor for stroke and chronic kidney disease [7,8]. Despite the casual relationship with some diseases above still being controversial, the epidemiological studies have provided much evidence for these associations. Due to the high prevalence and the serious adverse health outcomes caused by hyperuricemia, prevention and control of hyperuricemia have become more important than before.

Previous studies have identified individual risk factors for the incidence of hyperuricemia all over the world [2,10,11,12,13]. However, to our knowledge, a prediction model to establish an individual’s absolute risk of hyperuricemia is unavailable. Thus, in this study, we have developed gender-specific risk prediction models for hyperuricemia using a large population-based cohort in China, with the aim of predicting the incidence of hyperuricemia and preventing more adverse health outcomes associated with hyperuricemia.

## 2. Methods

### 2.1. Study Population

**Figure 1:**
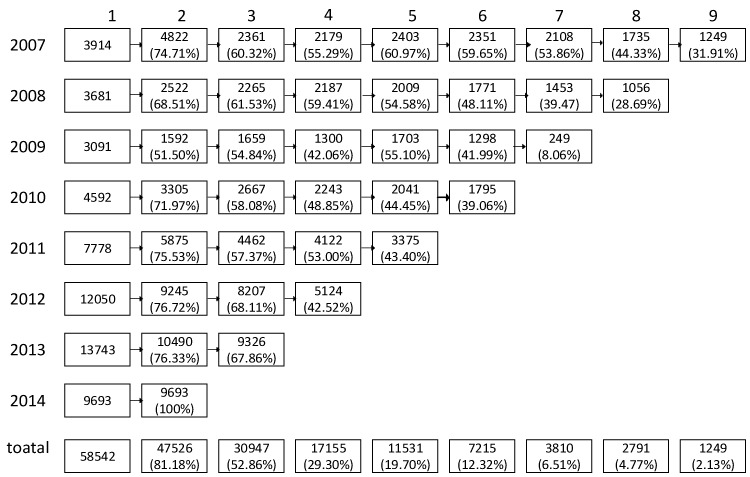
The samples of repeated surveys at each year.

This prospective cohort study was based on the database from large sample multicenter longitudinal health management cohorts in Shandong province which were the health information of participants who conducted the annual health examination in the centers. Participants without hyperuricemia in the first check-up at the year of 2007, 2008, 2009, 2010, 2011, 2012, 2013, 2014 and had no major biomarkers missing aged 20–80 years old were included in the baseline of prospective cohort study respectively. Figure 1 showed the total 58542 participants having at least two repeated health check-up within nine years (January 2007 to December 2015), and the samples of repeated surveys each year. The median follow-up year in our study was 2.5 years. This study was approved by the Ethics Committee of the School of Public Health (20140322), Shandong University. Written informed consent was obtained from all participants.

### 2.2. Biomarkers Selection and Measurements

We selected 10 anthropometric and blood biochemical biomarkers from routine health check-up data by reviewing previous studies which are associated with hyperuricemia and are readily available in clinical practice and precisely measured. They were age, body mass index (BMI), systolic blood pressure (SBP), triglycerides (TG), total cholesterol (CHOL), high-density lipoprotein cholesterol (HDL-C), low-density lipoprotein cholesterol (LDL-C), serum uric acid (SUA), serum creatinine (SCr), alanine aminotransferase (ALT). Height and weight were measured on participants wearing light clothing without shoes, and BMI was calculated as weight (kg) divided by squared height (m^2^). SBP and DBP were measured on right upper arm with participants in sitting position after 5 min rest. Peripheral blood samples were obtained in the morning after a 12 h fast to measure the following blood biochemical biomarkers: TG, CHOL, HDL-C, LDL-C, SUA, ALT. All lab tests were conducted by certified experimental specialist using standard protocols at hospital’s Department of Laboratory.

### 2.3. Outcomes

The primary outcome of the present analysis was hyperuricemia. Hyperuricemia was defined as first-ever development of hyperuricemia. The criteria for diagnosis of hyperuricemia was defined as a serum uric acid level 420 µmol/L in males and 360 µmol/L in females [14].

### 2.4. Statistical Analysis

Descriptive statistics were conducted for 58,542 participants at baseline. Student’s *t* test and nonparametric Wilcoxon test were used to detect the statistical significances for 10 biomarkers between two status of hyperuricemia in both males and females, and the Chi-square test was conducted to detect the difference in the incidence rate of hyperuricemia between two status of hyperuricemia in both males and females.

To develop the risk prediction models, we used 10 biomarkers from earlier reports as the potential predictors, we firstly conducted age-adjusted model for each 10 known biomarkers, then we developed multivariate Cox proportional hazards regression models using the biomarkers which were significant in the age-adjusted models for both males and females, the variable selection method of the multivariate Cox proportional hazards regression models was stepwise selection method. Cox proportional hazards regression models was used to estimate the relative risk (and corresponding 95% confidence intervals (CIs)) of hyperuricemia for each of the potential risk predictors. The probability of developing hyperuricemia within *t* years (*t* = 3) for an individual with covariate values *x* = (*x*_1_,…, *x_i_*) for *i* risk factors can be estimated using the following equation:

$$\mathrm{Risk estimate}=1-S_{0}{\left(t\right)}^{\exp(\sum^{\text{​}}\beta x-\sum^{\text{​}}\beta \bar{x})}$$

*S*_0_(*t*) is the mean survival probability at time *t* (e.g., *t* = 3 years) for an individual whose covariate values are all at a mean level, *β* are the estimated coefficients from the Cox proportional hazard model, *x* are the individual’s values on risk factors and $\bar{x}$ are the means of the risk factors. The probability of developing hyperuricemia for any set of covariate values can be estimated once *S*_0_(*t*) and *β* are obtained.

We conducted the internal validation of the prediction model and obtained a bias corrected estimate of the AUC using a 10-fold cross-validation procedure.

For both training set and 10-fold cross-validation, Receive Operate Characteristics curve (ROC curve) analysis was conducted to evaluate the discrimination of the models for both males and females [15]. The discrimination of a prediction model is the ability that indicates how well the model separates patients who experienced the event of interest from those who did not at three years. The area under the ROC curve (AUC) together with sensitivity, specificity, 95% Confidence Interval and cut-off of *p*-value was calculated by MedCalc software [16].

All statistical tests were two-sided with a type I error of 0.05, and *p*-value < 0.05 were considered statistically significant. Statistical analysis was carried out using SAS9.4 software (SAS Institute Inc., Cary, NC, USA).

## 3. Results

### 3.1. Hyperuricemia Incidence and Baseline Characteristics

**Figure 2:**
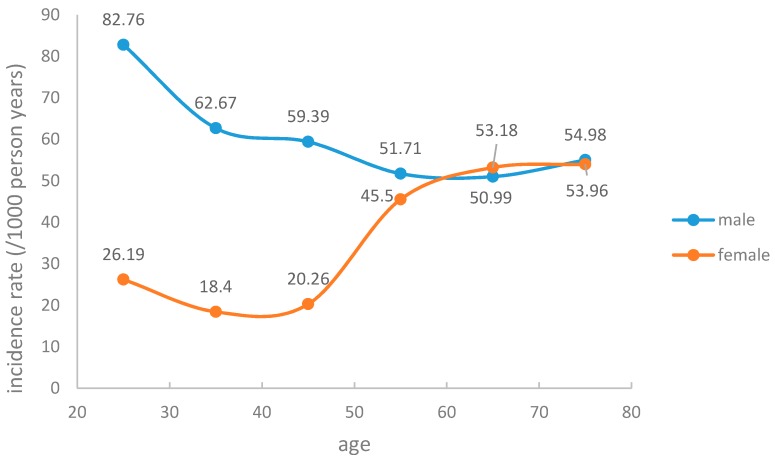
The incidence of hyperuricemia for both males and females.

For the cohort follow-up from 2007 to the end of 2015, the total number of person years of follow-up was 143,841 personyears (86,422 personyears for males and 57,295 personyears for females), with a median of 2.5 years of follow-up. The mean (SD) ages for males and females were 44 (13.36) and 42 (12.61) years, respectively. At the end of the follow-up period for 58,542 subjects, 7139 incident hyperuricemia cases (5585 males and 1554 females) were diagnosed, resulting in incidence rates of 64.62/1000 and 27.12/1000 personyears for males and females, respectively. Figure 2 shows the incidence rate varied with age; the blue trend line represents males and the red trend line represents females. From Figure 1 we can see that males and females had very different incidence rate tendencies that varied with age. There was a rough tendency for a decrease with the increase of age in males. However, the tendency in females was the opposite, with the incidence rate of hyperuricemia roughly increased with the increase of age. In addition, before the age of 60 years, the incidence rate of hyperuricemia in males was higher than in females, but males had a downtrend while females had an uptrend. After 60, the circumstance changed, with the trends of males and females varying almost the same with age.

Table 1 shows the baseline characteristics by the incident hyperuricemia status (hyperuricemia and non-hyperuricemia) for both males and females. For males, all 10 variables were significantly different between hyperuricemia and non-hyperuricemia, among which only age and HDL-C were lower in hyperuricemia than in non-hyperuricemia, and the others were all higher in hyperuricemia than in non-hyperuricemia. For females, all 10 variables were significantly different and only HDL-C was lower in hyperuricemia than in non-hyperuricemia, and the others were all higher in hyperuricemia than in non-hyperuricemia.

### 3.2. Construction of Prediction Models

Risk factors that were associated with hyperuricemia (*p* < 0.05) in the Cox regression model adjusted for age were eligible for inclusion in the final multivariable model. We developed gender-specific prediction models to estimate the three-year risk of hyperuricemia for both males and females. For males, the final multivariable model included age, SBP, BMI, and SUA. For females, the final multivariable model included BMI, SBP, SUA, and CHOL. Table 2 shows sample characteristics and the age-adjusted Cox regression model and Table 3 shows the *β* coefficient of the final prediction models for both males and females.

### 3.3. Evaluation of Prediction Models

**Figure 3:**
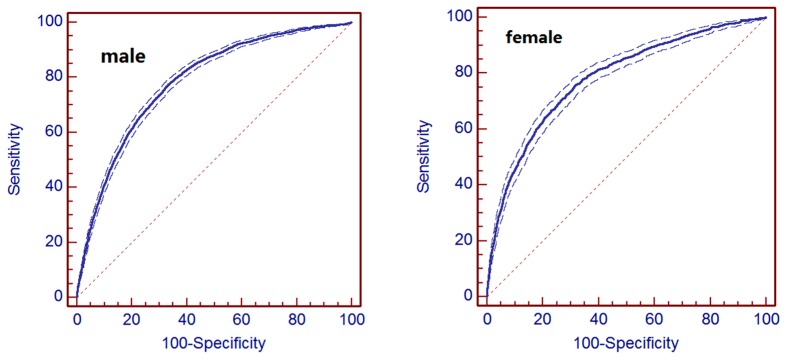
ROC curve for prediction of metabolic syndrome.

The receiver operating characteristic curve analysis was performed to evaluate the discrimination ability of the developed models. Figure 3 shows the results of the Receiver Operating Characteristic (ROC) analysis to predict the three-year risk of hyperuricemia using the developed models. It indicated that the C-statistics were 0.783 (95% confidence interval (CI), 0.779–0.786) for males and 0.784 (95% CI, 0.778–0.789) for females. After 10-fold cross-validation, the C-statistics were 0.782 for males and 0.783 for females.

The dotted plots stand for the 95% confidence interval. Males: Area under the ROC curve (AUC) is 0.783; standard error is 0.00459; 95% confidence interval is 0.777 to 0.786; Z statistic is 68.764; significance level *p* is (Area = 0.5) 0.0001. The point with the highest accuracy showed a sensitivity of 73.2 and a specificity of 69.7 under the cut-off of 0.1851. Females: Area under the ROC curve (AUC) is 0.784; standard error is 0.00851; 95% confidence interval is 0.778 to 0.789; Z statistic is 40.225; significance level *p* is (Area = 0.5) 0.0001. The point with the highest accuracy showed a sensitivity of 69.2 and a specificity of 74.2 under the cut-off of 0.0825.

## 4. Discussion

In this paper, gender-specific risk prediction models of hyperuricemia were developed using data obtained from a prospective cohort study of urban Han Chinese adults. The risk prediction models demonstrated good performance with high C statistics in both derivation and validation of the models. The models can be used for primary physicians to identify risk groups for hyperuricemia at an early stage and to give a warning for risk factor management of hyperuricemia after a routine health check-up. Consequently, people with high risk factors could be motivated to pay attention to their current health status and perform some proper management to lower the probability of developing hyperuricemia and potentially reduce the adverse outcomes associated with hyperuricemia such as gout, metabolic syndrome, cardiovascular disease, etc.

In the present study, the incidence rate of hyperuricemia was higher among males (61.51/1000 person years) than females (27.10/1000 person years). Hyperuricemia incidence rates changed with age; however, males had a downtrend while females had a rough uptrend. The reasons contributing to the obvious different trends of hyperuricemia incidence rates between males and females are complicated, perhaps because of hormonal changes during menopause in females or lifestyle changes (including dietary habits and smoking habits, etc.) in males. During youth, males are at high risk of developing hyperuricemia, which may result from unhealthy lifestyle habits such as the meat-based meals, drinking too much, etc. The bad habits may be reduced and result in the downtrend incidence rate with the increase of age. On the contrary, the female incidence rate decreases with the increase of age, which may result from hormonal changes; our study showed that in females between 40 and 60 years old, the incidence rate rises steeply, at which point most females are in the menopause transition years. After menopause, hyperuricemia occurs more frequently due to estrogen deficiency [17,18]. This phenomenon needs to be further researched.

The final predictors we used in our prediction models were very simple and readily accessible anthropometric and blood biochemical biomarkers including age, BMI, SBP, SUA, and TG. These predictors were all proved to be strongly associated with the incidence of hyperuricemia in previous studies. A previous analysis on Chinese people demonstrated that SUA levels of male participants reached a peak between 13 and 18 years of age and decreased after 18 years, while in females, SUA levels were stable before 18 years and increased in the mid to older age group [19]; this is similar to the results of our study, proving that age is associated with hyperuricemia. Cohort studies in China, Korea, America and Japan showed that BMI, SBP, and TG are associated with hyperuricemia [2,10,11,12]. A Mendelian randomization study verified the effect of BMI on hyperuricemia [20]. Our results not only confirmed these risk factors for an increased risk of hyperuricemia but also developed very simple prediction models which are specifically designed for the urban Han Chinese adults with these risk factors.

The strengths of this cohort study include the large sample size of the cohort, which can minimize the risk of bias. In addition, the predictors of our models are all readily accessible anthropometric and blood biochemical biomarkers which are more accurate than questionnaire variables to avoid recall bias.

The limitations of our study should be mentioned. Firstly, the participants included in our study were all urban Han Chinese adults, so our prediction model may not be generalizable to other populations. Therefore, external validations of the risk prediction model are needed. Secondly, our results are all based on one-time measurement, which may not reflect the status of the participants accurately and may be overestimate the incidence rate of hyperuricemia. Thirdly, interval censoring of the event which may affect the accuracy of the results must exist.

## 5. Conclusions

In conclusion, we have developed and validated gender-specific prediction models in an urban Han Chinese adult population. As our study has a large sample size, including a wider age range, the estimates from our prediction model were more stable, as demonstrated by the 10-fold cross-validation. We have constructed two prediction models for predicting the three-year incidence of hyperuricemia for both males and females only using simple and facile routine anthropometric and blood biochemical biomarkers. These simple models could help in identifying high-risk groups and encouraging these people to pay attention to their health status, take timely action, and finally prevent gout and other adverse health outcomes.

## Figures and Tables

**Table 1:** Baseline characteristics by incident hyperuricemia status of males and females.

Variables	Males		*p*-Value	Females		*p*-Value
	HUA	Non-HUA		HUA	Non-HUA	
	(*n* = 5581)	(*n* = 29,395)		(*n* = 1554)	(*n* = 22,008)	
Age (years)	41.69 ± 12.91	43.85 ± 13.42	<0.0001	45.48 ± 14.59	41.21 ± 12.41	<0.0001
SBP (mmHg)	132.6 ± 17.37	130.4 ± 17.32	<0.0001	127.7 ± 21.18	120.5 ± 18.11	<0.0001
BMI (kg/m^2^)	25.98 ± 3.12	24.91 ± 3.23	<0.0001	24.35 ± 3.57	22.61 ± 3.21	<0.0001
SUA (µmol/L)	369.4 ± 40.94	320.5 ± 52.86	<0.0001	294.7 ± 45.21	245.0 ± 47.89	<0.0001
CHOL (mmol/L)	4.97 ± 0.93	4.83 ± 0.90	<0.0001	5.06 ± 1.02	4.75 ± 0.93	<0.0001
TG (mmol/L)	1.91 ± 1.56	1.54 ± 1.26	<0.0001	1.41 ± 0.92	1.07 ± 0.77	<0.0001
HDL-C (mmol/L)	1.31 ± 0.31	1.35 ± 0.32	<0.0001	1.47 ± 0.33	1.54 ± 0.35	<0.0001
LDL-C (mmol/L)	2.95 ± 0.73	2.86 ± 0.72	<0.0001	2.95 ± 0.81	2.68 ± 0.75	<0.0001
SCr (µmol/L)	71.25 ± 13.63	70.04 ± 14.91	<0.0001	54.35 ± 10.61	52.97 ± 11.18	<0.0001
ALT (U/L)	30.31 ± 23.31	26.62 ± 21.95	<0.0001	20.47 ± 13.89	17.30 ± 14.86	<0.0001

values are means ± standard deviations.

**Table 2:** Sample characteristics and Cox proportional hazards model (adjusted for age) for three-year risk of hyperuricemia.

Variables	Sample Characteristics	HR (95% CI)	*p*-Value
*Males*			
Age (years)	43.50 (13.36)	0.990 (0.988–0.992)	<0.0001
SBP (mmHg)	130.75 (17.34)	1.009 (1.008–1.011)	<0.0001
BMI (kg/m^2^)	25.08 (3.23)	1.101 (1.093–1.109)	<0.0001
SUA (µmol/L)	328.35 (54.18)	1.022 (1.022–1.023)	<0.0001
CHOL (mmol/L)	4.86 (0.91)	1.156 (1.124–1.189)	<0.0001
TG (mmol/L)	1.60 (1.32)	1.105 (1.093–1.118)	<0.0001
HDL-C (mmol/L)	1.35 (0.32)	0.741 (0.681–0.806)	0.0004
LDL-C (mmol/L)	2.87 (0.72)	1.217 (1.171–1.261)	<0.0001
SCr (µmol/L)	70.23 (14.72)	1.006 (1.005–1.006)	<0.0001
ALT (U/L)	27.21 (22.21)	1.004 (1.003–1.004)	<0.0001
*Females*			
Age (years)	41.49 (12.61)	1.025(1.021–1.029)	<0.0001
SBP (mmHg)	120.93 (18.42)	1.014 (1.011–1.017)	<0.0001
BMI (kg/m^2^)	22.72 (3.26)	1.095 (1.085–1.106)	<0.0001
SUA (µmol/L)	248.28 (49.28)	1.023 (1.022–1.024)	<0.0001
CHOL (mmol/L)	4.77 (0.94)	1.160 (1.098–1.224)	0.0034
TG (mmol/L)	1.08 (0.78)	1.161 (1.130–1.193)	<0.0001
HDL-C (mmol/L)	1.53 (0.35)	0.653 (0.565–0.754)	0.0019
LDL-C (mmol/L)	2.70 (0.76)	1.320 (1.237–1.410)	<0.0001
SCr (µmol/L)	53.06 (11.15)	1.005 (1.003–1.006)	0.0209
ALT (U/L)	17.51 (14.82)	1.004 (1.003–1.006)	<0.0001

Data are mean (SD); HR: Hazard ratio, CI: confidence interval.

**Table 3:** Multivariable-adjusted Cox proportional hazards regression coefficients for three-year risk of hyperuricemia.

Variables	*β* Coefficient	SE	HR (95% CI)	*p*-Value
*Males*				
Age (years)	−0.00891	0.0012	0.991 (0.989–0.993)	<0.0001
SBP (mmHg)	0.00445	0.0010	1.004 (1.003–1.006)	<0.0001
BMI (kg/m^2^)	0.03744	0.0054	1.042 (1.033–1.051)	<0.0001
SUA (µmol/L)	0.02095	0.0004	1.022 (1.021–1.022)	<0.0001
*Females*				
BMI (kg/m^2^)	0.04934	0.0071	1.051 (1.036–1.065)	<0.0001
SBP (mmHg)	0.00523	0.0016	1.005 (1.003–1.008)	<0.0001
SUA (µmol/L)	0.02196	0.0006	1.022 (1.021–1.023)	<0.0001
TG (mmol/L)	0.04633	0.0235	1.047 (1.000–1.097)	0.0483

*S*_0_ = 0.924 (three-year baseline survival) for males and *S*_0_ = 0.971 (three-year baseline survival) for females. *β* values are expressed per one unit increase for the continuous variables.

## Abbreviations

BMI	body mass index
SBP	systolic blood pressure
TG	triglycerides
CHOL	total cholesterol
HDL-C	high-density lipoprotein cholesterol
LDL-C	low-density lipoprotein cholesterol
SUA	serum uric acid
SCr	serum creatinine
ALT	alanine aminotransferase
HUA	Hyperuricemia
CI	Confidence interval
